# Supplementary material for: Cryo-EM structure and evolutionary history of the conjugation surface exclusion protein TraT
Source: Nat Commun. 2025 Jan 14;16:659. doi: 10.1038/s41467-025-55834-w (PMC11733297; doi:10.1038/s41467-025-55834-w)
Supplement: Supplementary file 1 — Supplementary information [file 41467_2025_55834_MOESM1_ESM.pdf]

a

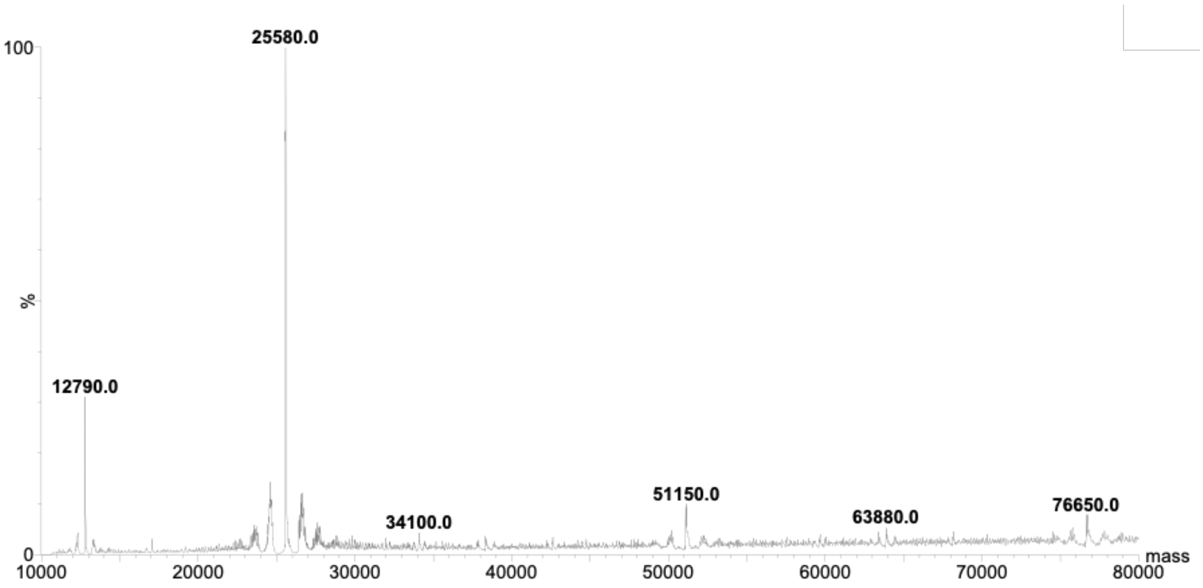

b

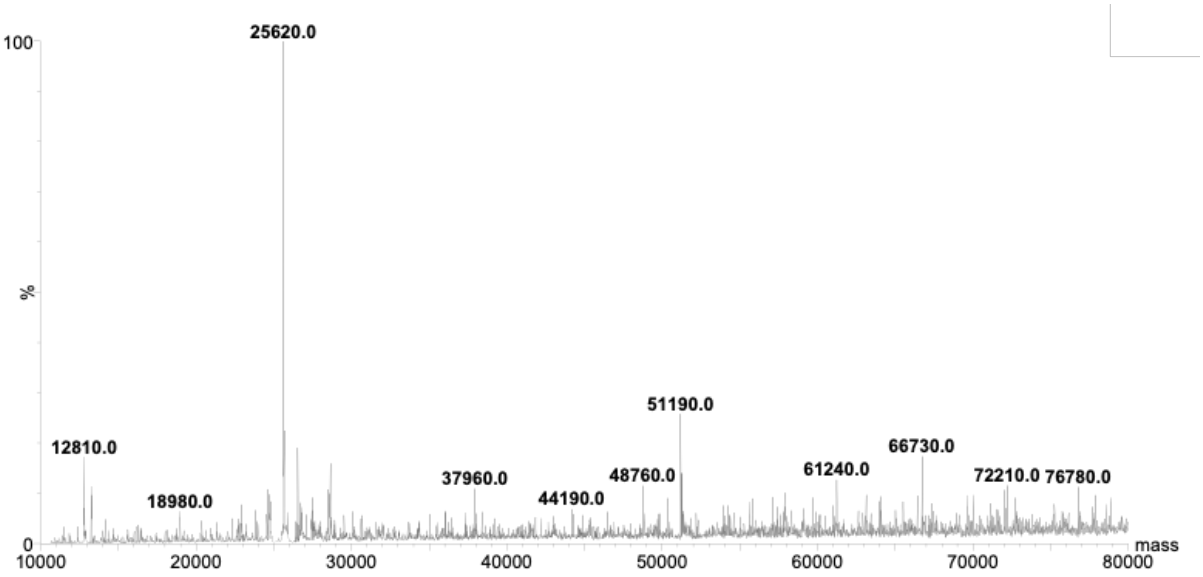

Supplementary Fig. 1. Mass spectrometry analysis of the (a) $TraT_{pKpQIL}$ and (b) $TraT_F$ proteins. The predicted molecular weight for the mature $TraT_{pKpQIL}$ and $TraT_F$ are 23,735.81 Da and 23953.13, respectively; the constructs carry an additional 1014.06 Da from cloning. The additional mass can be attributed to the DAG and PA modifications with an average molecular weight of around 300-700 Da and 256 Da, respectively. The DAG acyl chain length differs by 200 Da between the two proteins.

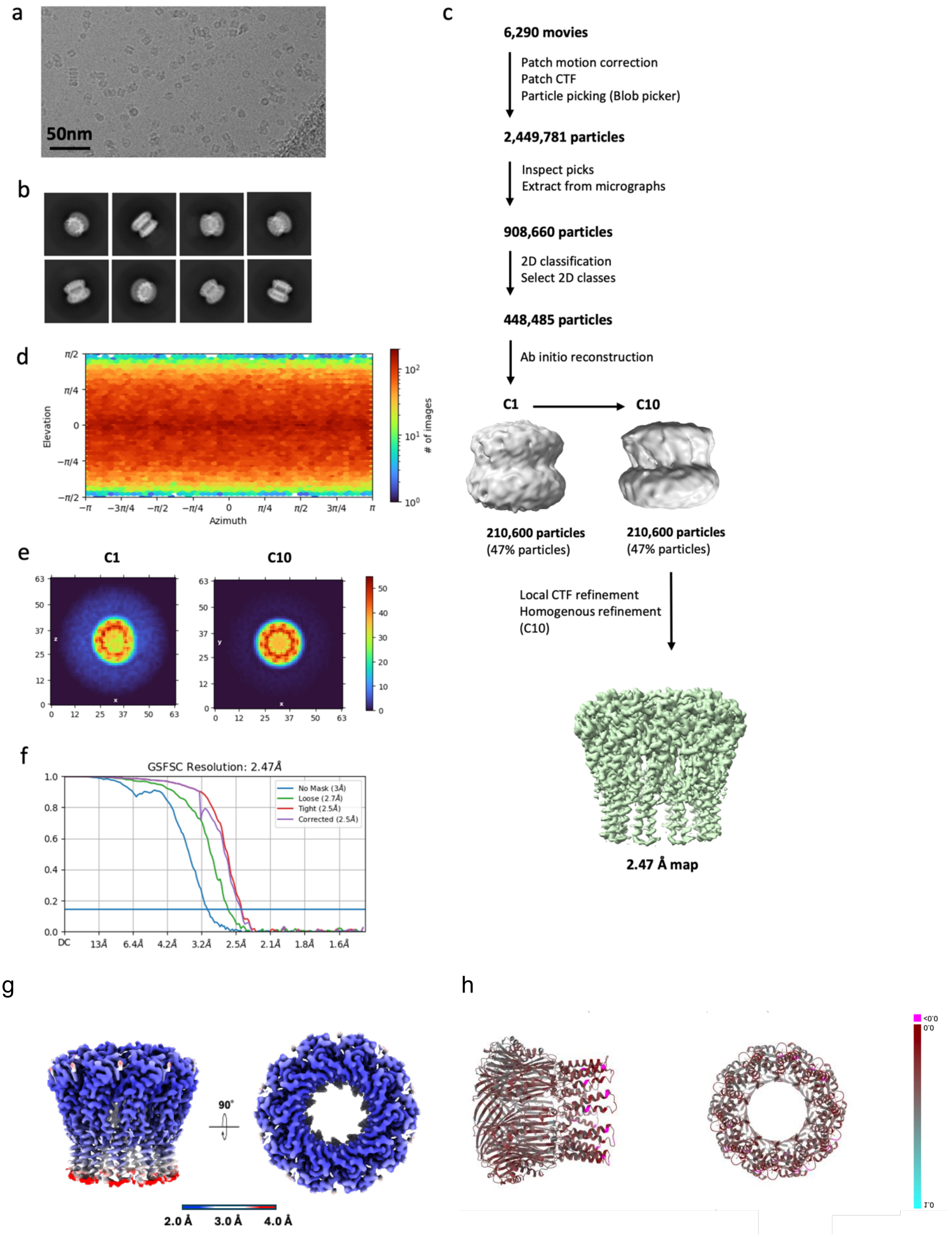

Supplementary Fig. 2. Electron microscopy analysis of $TraT_{pKpQIL}$. (a) Representative electron micrograph (dose-weighted averaged movie) of $TraT_{pKpQIL}$. (b) A selection of representative 2D class-averages. (c) Data processing pipeline. (d) Euler angle distribution plot. (e) Top view Image projection of the ab initio reconstruction of $TraT_{pKpQIL}$ using 100% of

the particles, shown with C1 and C10 symmetry applied**.** The colour bar shows the density levels in the volume. (f) GSFSC curve calculated using two independent half-maps (0.143). (g) Local resolution as estimated by cryoSPARC. (h) Model with each residue coloured according to its Q-score.

a

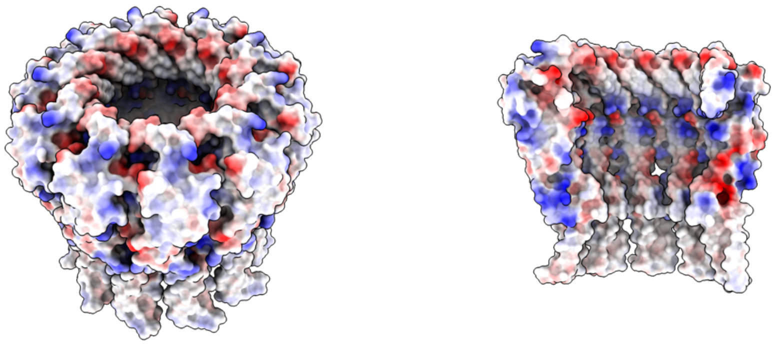

b

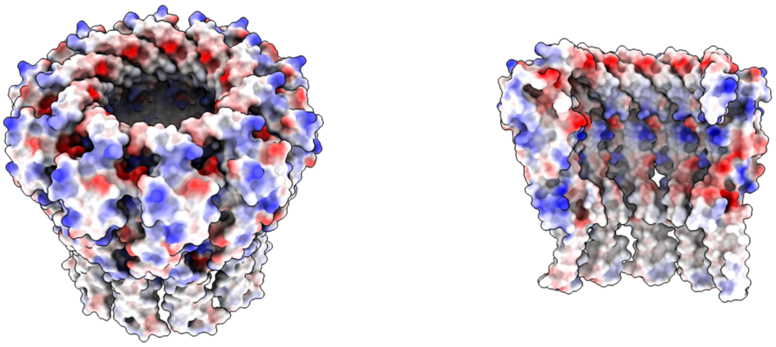

Supplementary Fig. 3. Electrostatic surface potential of (a) TraT$_{pKpQIL}$ and (b) TraT$_{F}$. (left panel) electrostatic map for the whole decamer. (right panel) A slice view of the central cavity displays varied charge distribution with a prominent negatively and positively charged belt inside the β-barrel domain; five protomers have been omitted for clarity. Both TraT$_{pKpQIL}$ and TraT$_{F}$ display the same charge distribution.

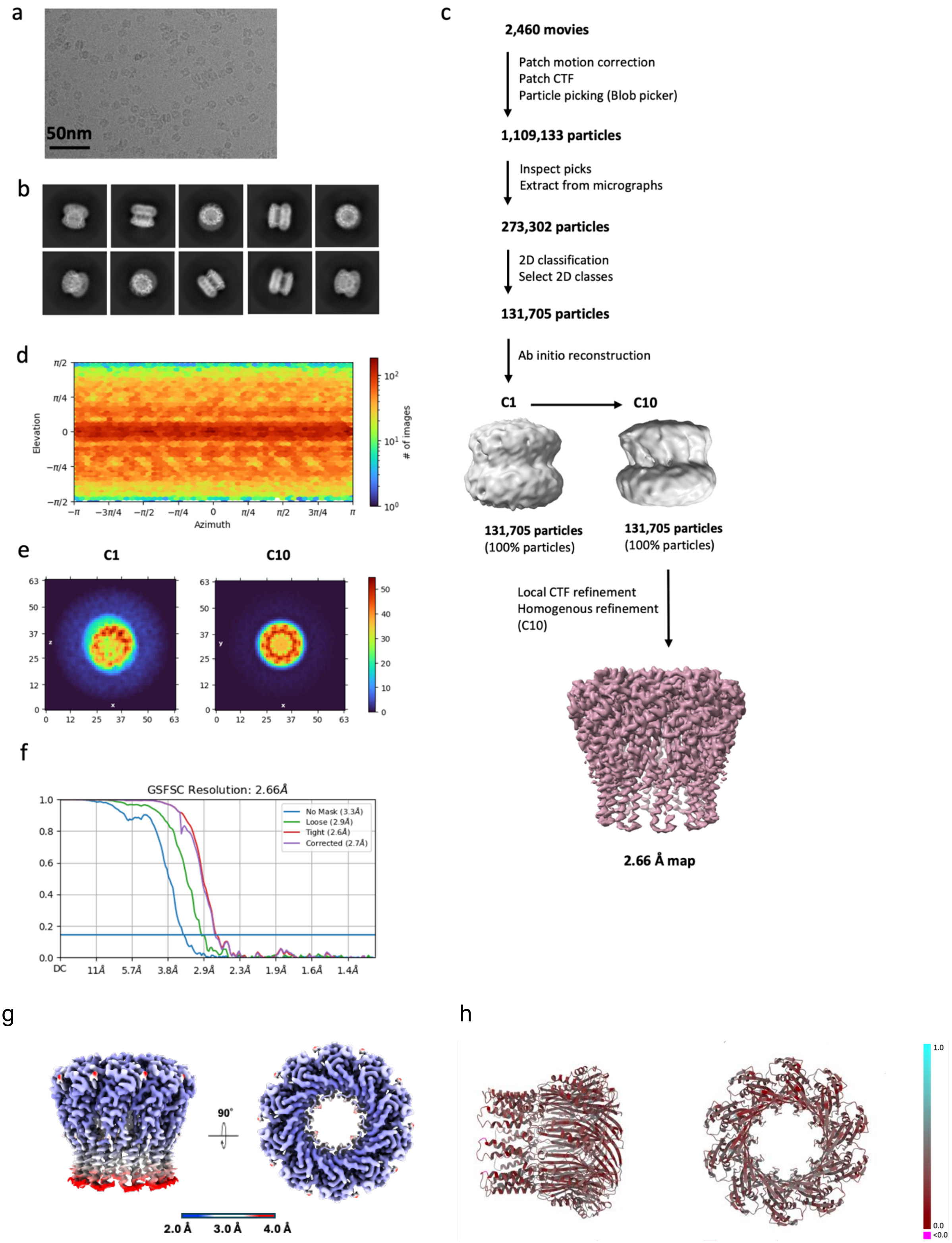

Supplementary Fig. 4. Electron microscopy analysis of $TraT_F$. (a) Representative electron micrograph (dose-weighted averaged movie) of $TraT_F$. (b) A selection of representative 2D class-averages. (c) Data processing pipeline. (d) Euler angle distribution plot. (e) Top view Image projection of the ab initio reconstruction of $TraT_F$ using 100% of the particles, shown

with C1 and C10 symmetry applied**.** The colour bar shows the density levels in the volume. (f) GSFSC curve calculated using two independent half-maps (0.143). (g) (g) Local resolution as estimated by cryoSPARC. (h) Model with each residue coloured according to its Q-score.

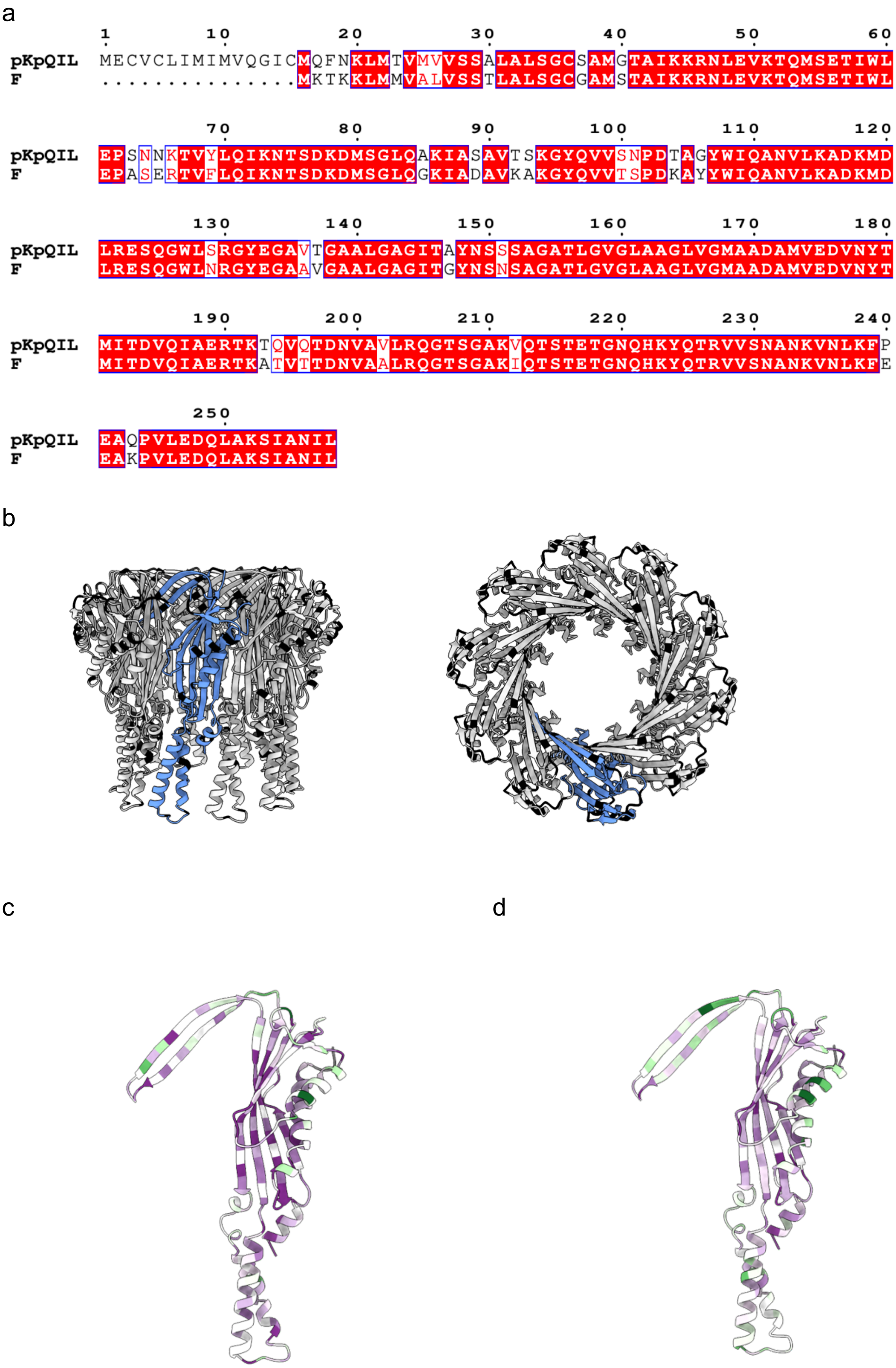

Supplementary Fig. 5. Sequence analysis. (a) Sequence alignment of $\text{TraT}_{\text{pKpQIL}}$ (accession ID: ARQ19738.1) with TraT_{F} (accession ID: WP_00085042.1) shows the distribution of amino acid differences between the two proteins. Columns with conserved residues are coloured

red with white lettering and columns with similar residues (similarity score <0.7) are outlined in blue boxes with red lettering. (b) Amino acid differences (shown in black) between $TraT_{pKpQIL}$ and $TraT_F$ mapped onto the $TraT_{pKpQIL}$ decamer; (left panel) side view and (right panel) top view. Most differences are found at the exterior of the β-sandwich domain suggesting a possible role in specificity. The decameric $TraT_{pKpQIL}$ is shown as grey cartoons and one protomer is coloured blue. (c) The conservation of the plasmid TraTs have been mapped onto the $TraT_{pKpQIL}$ structure. (d) The conservation of the chromosomal TraTs have been mapped onto the $TraT_{pKpQIL}$ structure. The least conservation is found at the α2 and β-hairpin motif. The conservation increases from green to purple.

Supplementary Table 1. Data collection and refinement statistics.

	$TraT_{pKpQIL}$	$TraT_F$
**Data collection and processing**		
Microscope	Titan Krios	Titan Krios
Magnification	165000 x	130000 x
Voltage (kV)	300	300
Electron dose ($e^-/Å^2$)	40 $e^-/Å^2$	40 $e^-/Å^2$
Detector	Falcon 4i	K3
Defocus range (-µm)	0.9 – 3.0	0.9 – 3.0
Pixel size (Å)	0.723	0.653
Symmetry imposed	C10	C10
Micrographs (no.)	6290	2460
Initial particle images (no.)	2449781	1109133
Final particle images (no.)	210600	131705
Global map resolution (Å)	2.47	2.66
FSC threshold	0.143	0.143
**Refinement**		
Model resolution (Å)<br>FSC threshold	2.5<br>0.143	2.7<br>0.143
Map sharpening *B* factor ($Å^2$)	-20	-35
Model composition<br>Non-hydrogen atoms Protein residues<br>Ligands<br>DAG	 2260<br><br>10	 2250<br><br>-
Mean B factors ($Å^2$)<br>Protein<br>DAG	 112.56<br>144.54	 136.08<br>-
R.m.s. deviations<br>Bond lengths (Å)<br>Bond angles (°)	 0.006<br>0.634	 0.004<br>0.510
**Validation**		
MolProbity score	2.16	2.08
Clash score	7.35	6.26
Ramachandran plot<br>Favored (%)<br>Allowed (%)<br>Disallowed (%)	 94.96<br>5.04<br>0	 95.52<br>4.48<br>0

Supplementary Table 2. Bacterial strains used for conjugation studies.

Strain	Description	Resistance	Source
***K. pneumoniae* strains**			
ICC8001	*K. pneumoniae* parental strain (WT)	Rif	Low WW, *et al* [1]
**Donor strains**			
GFP-D	ICC8001 carrying the pKpGFP parental reporter plasmid (pKpQIL tagged with P*lac-sfGFP* at the disrupted *aadA* gene).	Ery	Low WW, *et al* [1]
GFP-DD	ICC8001 carrying a derepressed variant of pKpGFP; pKpGFP-D	Ery	Low WW, *et al* [1]
**Recipient strains**			
pBAD-$traT_{pKpQIL}$	ICC8001 carrying the pBAD vector encoding $traT_{pKpQIL}$	Kan	This study
pBAD-$traT_{F}$	ICC8001 carrying the pBAD vector encoding $traT_{F}$	Kan	This study
pBAD-$traT_{C36S}$	ICC8001 carrying the pBAD vector encoding $traT_{C36S}$	Kan	This study
pBAD-$traT_{\Delta C36/\alpha 1}$	ICC8001 carrying the pBAD vector encoding $traT_{\Delta C36/\alpha 1}$	Kan	This study
pBAD	ICC8001 carrying the pBAD vector	Kan	This study

Supplementary References

1 Low, W. W. *et al.* Mating pair stabilization mediates bacterial conjugation species specificity. *Nat Microbiol* **7**, 1016-1027 (2022). https://doi.org/10.1038/s41564-022-01146-4
